# Fluorescence-Guided Surgery for Necrotizing Soft Tissue Infections: A Systematic Review

**DOI:** 10.7759/cureus.115431

**Published:** 2026-08-30

**Authors:** Rasmus Grønborg Bjærge, Luit Penninga

**Affiliations:** 1 Department of Gastrointestinal Surgery, Aalborg University Hospital, Aalborg, DNK; 2 Department of Clinical Medicine, Aalborg University Hospital, Aalborg, DNK

**Keywords:** fluorescence guided surgery, icg, indocyanine green, necrotizing fasciitis, necrotizing soft-tissue infection, nsti

## Abstract

Necrotizing soft tissue infections are life-threatening infections of soft tissue that are treated with rapid surgical debridement when the diagnosis is suspected. Necrotic tissue is removed until vital tissue is identified, guided by the surgeon's vision and hands. Fluorescence-guided surgery is increasingly popular for assessing tissue perfusion. This surgery may have the potential to improve the diagnosis and treatment of necrotizing soft tissue infections. A systematic review on fluorescence-guided surgery for necrotizing soft tissue infections was conducted according to the PRISMA guidelines and Cochrane methodology. A study protocol was prospectively registered on PROSPERO (registration number: CRD420261348991). A comprehensive search was performed across PubMed, Embase, the Cochrane Library, Scopus, Web of Science, ClinicalTrials.gov, and the WHO Clinical Trials Registry Platform. Furthermore, a bibliographic search of relevant literature was conducted. A total of 2377 records were identified and screened. We identified two case studies, including a total of three patients. Both studies found the technique to be feasible in the surgical debridement of necrotizing soft tissue infections, with no adverse events. The main limitation of this review is the limited literature on fluorescence-guided surgery for necrotizing soft tissue infections identified, with only two case studies included. Both reports showed potential benefits in surgical management; however, further studies are urgently needed. This systematic review found a limited number of studies on indocyanine green (ICG) fluorescence-guided surgery in patients with necrotizing soft tissue infections, and the identified evidence was insufficient to draw firm conclusions. Both reports indicate the method’s feasibility, with a potentially greater assessment of tissue perfusion.

## Introduction and background

Necrotizing soft tissue infections (NSTIs) are life-threatening, rapidly evolving monomicrobial or polymicrobial infections of the skin, subcutaneous layer, fascia, and muscle, predominantly caused by bacteria. NSTIs have a relatively low prevalence but carry a high risk of mortality and severe morbidity. The pathophysiology involves the production of exotoxins leading to extensive tissue destruction as well as small-vessel thrombosis, inevitably systemic effects resulting in, organ failure, septic shock, and death. The infections necessitate immediate, aggressive surgical debridement as well as intravenous antibiotic and fluid treatment. Diagnosing NSTIs is challenging due to non-specific symptoms (e.g., erythema, edema, and disproportionate pain), elevated inflammatory markers, and not necessarily an obvious bacterial entry site, which can easily be mistaken for a less severe infection or inflammation. Physical examination, clinical tools (e.g., LRINEC score), and radiological imaging are routinely performed; however, their limited sensitivity and specificity pose a challenge for real-time diagnosis [1,2]. Late clinical findings, such as bullae, ecchymoses, or hypotension, are more specific; nevertheless, valuable time has already been lost by the time these signs are present. Once suspicion of NSTI arises, immediate surgical intervention is of utmost importance. Microbial culturing, biopsy, and histological examination are standard practices, but do not yield immediate diagnostic information. Perioperative findings of “dishwasher-like” gray exudate, fragile soft tissue that easily separates with the touch of a finger, and diminished bleeding are diagnostically significant, and the surgical treatment requires removing all necrotic material until the remaining tissue is distinctly viable and bleeding. The surgical procedure often involves the removal of important structures such as major nerves and blood vessels, and occasionally amputations. The removal of necrotic tissue relies solely on the eyes and hands of the surgeon [1,3]. It is generally recommended to perform an operative reassessment every six hours after initial surgery and to remove further necrotic tissue [4]. The average number of surgical reassessments in patients with NSTI is three [5]. 

Fluorescence-guided surgery is gaining ground and becoming increasingly popular due to its visualization-augmenting properties, which improve surgical efficacy and safety [6]. The fluorophore indocyanine green (ICG) is an extensively studied agent with more than 60 years of FDA approval for liver function assessment, ophthalmology, and angiography [7]. Adverse events are rare, with anaphylaxis occurring in fewer than one in 10,000 cases [6]. With a peak spectral absorption at ~800 nm and emission of near-infrared wavelengths (~830 nm), it can be visualized with a near-infrared camera. ICG binds to albumin in the blood, and tissue perfusion can be visualized moments after administration. Additionally, ICG can be conjugated to target-specific probes and thereby visualize selected tissues, such as tumors [8]. A recent study showed promising results in real-time diagnostics of NSTI, showing accurate discrimination between cellulitis and NSTI (n = 12), as well as showing areas with decreased perfusion that were not identified under white light, and proposed the possibility of fluorescence-guided surgery in assisting in the removal of necrotic tissue in NSTI [7].

## Review

Methods

This review was conducted according to the Preferred Reporting Items for Systematic Reviews and Meta-Analyses (PRISMA) guidelines (https://www.prisma-statement.org) and the Cochrane methodology. A study protocol was prospectively registered on PROSPERO (registration number: CRD420261348991). A comprehensive search was conducted across PubMed, Embase, the Cochrane Library, Scopus, and Web of Science. Furthermore, a search for relevant studies on ClinicalTrials.gov and the WHO Clinical Trials Registry Platform was conducted. The search was performed on August 17, 2025. All studies were imported into Covidence (www.covidence.org) and checked for duplicates. Titles, abstract screening, and full-text review were performed by two researchers (RB and LP). Additional publications were identified through a bibliographic search of relevant articles. Two researchers conducted data extraction of the included articles. All publications were risk-assessed using JBI’s critical appraisal checklist for case reports (https://jbi.global/critical-appraisal-tools) [9], and an overall quality assessment was provided. As only case reports met the inclusion criteria, the pre-specified use of ROB-2 and ROBINS-I, as well as meta-analysis using RevMan (The Cochrane Collaboration, Oxford, UK), was not applicable. A consensus between both researchers resolved any disagreements. All studies on fluorescent guided surgery for the treatment of NSTI published in English were included. Given the expectation of limited literature on the topic of this systematic review, all study designs were included, including RCTs, cohort studies, case reports, trials, and case-control studies. The primary outcomes were mortality, serious adverse events, and quality of life. Secondary outcomes were surgical duration (primary and secondary procedures), number of surgical interventions, length of hospital stay, intensive care stay, and number of patients with amputations. 

Results

A total of 2377 records were identified through the database searches (Figure 1). Following title/abstract and full-text screening, one study [10] met the inclusion criteria. Three reports from the same study [7,11,12] were identified that examined the use of fluorescence as a diagnostic tool in patients with NSTI, utilizing methods relevant to the topic of this review. However, since no patients were treated with fluorescence-guided surgery, the reports were excluded. Bibliographical search of the above-mentioned literature identified one additional article that met the inclusion criteria [13]. A total of two studies were included, including a total of three patients. An overview of the included studies is shown in Table 1. Aka et al. [13] present a case of NSTI involving the left labia and the left gluteal fold. Following standard surgical removal of necrotic tissue at second look, a fluorescence perfusion assessment was performed, revealing additional areas of low perfusion, consistent with necrotic tissue, not detected under white light. These areas were successively removed. Subsequently, no further surgery was needed. The patient was discharged on the ninth postoperative day. Bongard et al. [10] present two cases of patients with presumed NSTI: one in the right volar forearm and one in the right supraclavicular fossa. Using sodium fluorescein to assess tissue perfusion, fluorescence measurements were performed and marked on the skin. Marking areas of low perfusion for removal and securing the surrounding flaps had sufficient perfusion, indicated by high fluorescence values. Both cases showed no further necrosis during the second-look operation. None of the patients in the included studies experienced serious adverse events, amputations, need for intensive care, or death.

**Figure 1:**
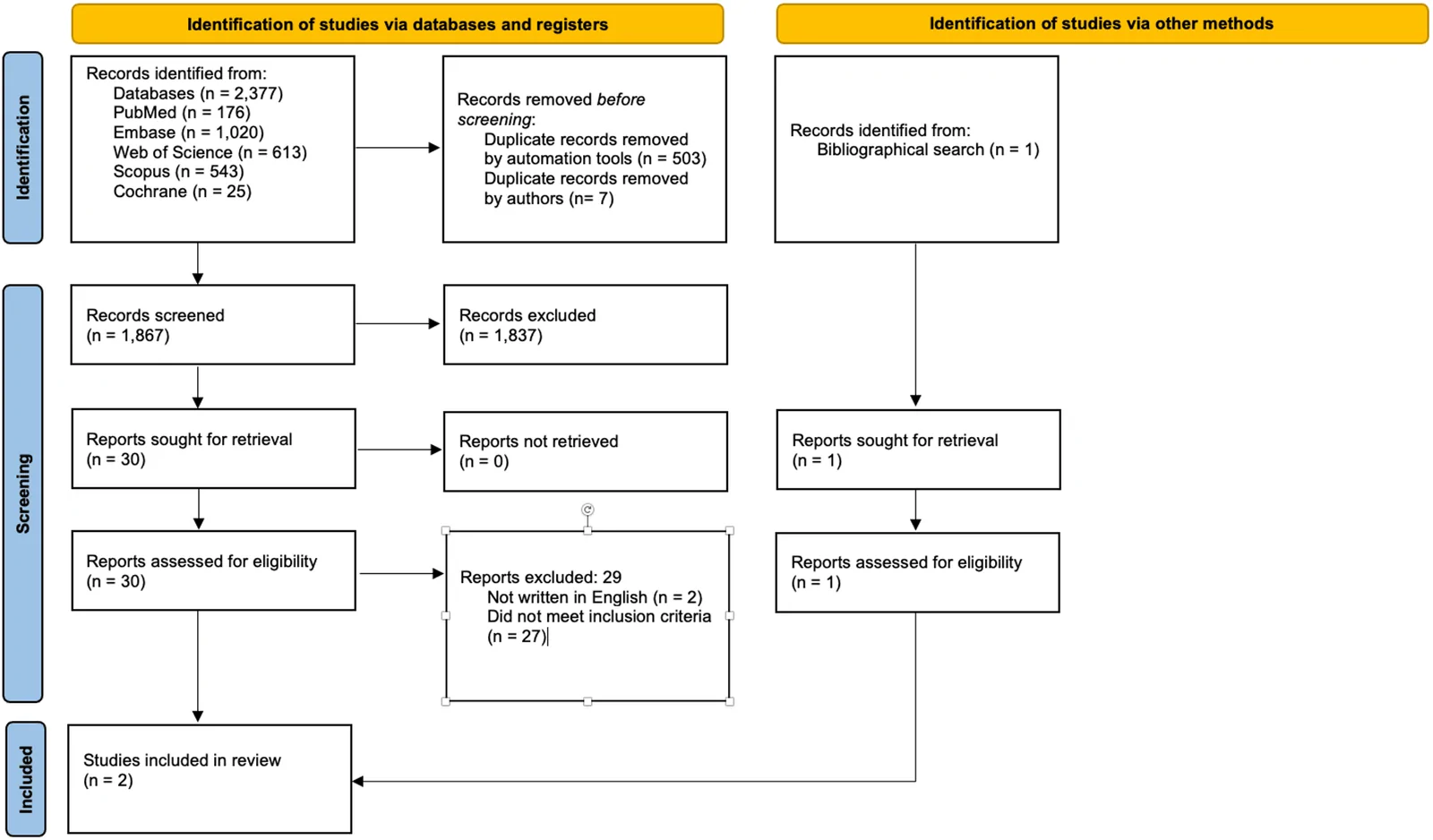
PRISMA 2020 flow diagram of the study selection process Reference: [14]

**Table 1:** Characteristics of included studies

Author, year	Design	Number of participants	Study period	Fluorophore, dosage	Imaging system	Outcome	Quality of the evidence
Bongard et al., 1985 [10]	Case report	2	Not reported	Sodium fluorescein, 1-2 mg/kg	Digital dermofluorometry	Successful removal of necrotic skin during initial debridement	Low quality
Aka et al., 2021 [13]	Case report	1	Not reported	Indocyanine green, dosage NA	Stryker SPY-PHI system	Showing non-viable areas of skin not visible under white light	Good quality

Discussion

This systematic review assessed the use of fluorescence-guided surgery for NSTIs. Limited literature was identified, with available data restricted to three patients across two case reports, one of which was published more than 40 years ago. Both reports found fluorescence guidance beneficial for the surgical management of NSTI; however, further studies are urgently needed. Ray et al. examined 14 patients suspected of NSTI using perfusion-based ICG fluorescence imaging and found signal voids corresponding to affected tissue, showing statistically significant differences in variables, such as lesion-to-background ratio, ingress slope, and maximum fluorescence intensity, between patients with NSTI and cellulitis (p-value < 0,05) [7]. These findings support the potential use of ICG fluorescence in the surgical management of NSTI. ICG fluorescence imaging to identify areas of low perfusion and tissue necrosis has been examined in other clinical contexts, where early detection of necrosis has been critical. A review by Green et al. examined the use of intraoperative ICG angiography in war-related traumatic and reconstructive cases [15]. War-related traumatic wounds are usually contaminated, and progressive tissue necrosis is frequent. The review found that ICG angiography lowered perfusion-related complications by enabling intraoperative identification of low-perfused tissue. Thus, resulting in further excision of distal tissue and changes in the placement of skin closure due to the risk of dehiscence, not detected before ICG fluorescence angiography was performed [15]. One study found that 47% of procedures were modified due to findings from ICG fluorescence angiography, concluding that the method had the highest prognostic power for predicting tissue necrosis [16]. Patients with peripheral arterial disease have also been subject to ICG fluorescence imaging. One study examined 10 patients with critical limb ischemia after amputations of a lower extremity were performed and showed the method could predict later necrosis in patients with perfusion deficits [17]. While ICG fluorescence angiography appears advantageous for assessing necrotic tissue in patients with NSTI, molecular-guided debridement might also be a viable tool for detecting necrosis. Patients with diabetic foot ulcers present the same challenges for surgeons in removing necrotic tissue, which requires surgeons to identify the affected tissue under white light. One pilot study examines fluorescent-guided debridement with ICG in complex with lipoprotein (ICG-LP), which binds to necrotic tissue. The study found ICG-LP useful for the debridement of “fresh” necrotic tissue, resulting in a significant reduction in the number of surgical debridements needed for infection control compared to standard debridement. The main limitation is reduced fluorescence in severe necrotic tissue, although this could be recognized with the naked eye [18]. The same technique could potentially be applied to patients with NSTI. Near-infrared spectroscopy is another method for assessing tissue perfusion by utilizing hemoglobin’s ability to absorb near-infrared light, whereas deoxygenated hemoglobin does not [19]. Near-infrared spectroscopy correlates well with transcutaneous PO2 measurements [19,20]. Evidence suggests that near-infrared spectroscopy might be a viable method for diagnosing NSTI. One study examined 234 patients suspected of soft tissue infections of the lower extremities; 19 patients had NSTI and 215 had cellulitis. At an oxygen saturation threshold of 70%, they found near-infrared spectroscopy to have 100% sensitivity and 97% specificity [21]. Other studies found similar results by determining the ratio of the affected and healthy leg [22,23]. Near-infrared spectroscopy also showed promising results for assessing healing capacity and the risk of dehiscence in patients with diabetic foot ulcers [20]. Near-infrared spectroscopy has various advantages, including non-invasiveness, affordability, ease of use, and continuous measurement. Limitations include sensitivity to competing conditions, such as systemic hypoxia or shock, and, regarding extremities, peripheral vascular disease, chronic venous insufficiency, and deep venous thrombosis [21-23]. Also, higher levels of skin melanin might affect measurements [20]. Many of the limitations apply to fluorescence-guided debridement and should be considered. Another limitation is that the aforementioned studies use comparable “healthy” tissue for assessment [20-23], which questions the method’s validity for NSTI of the truncus or anogenital region. The use of near-infrared spectroscopy in an operative setting is speculative. Still, its non-invasive nature and ease of handling could have a role in monitoring patients with NSTI after primary surgery.

An updated literature search conducted on August 15, 2026, covering publications from 2025 and 2026, identified no additional studies meeting the predefined inclusion criteria. However, Henderson et al. [24] recently reported the implementation of an ongoing prospective, multicenter observational study evaluating ICG fluorescence imaging in the diagnostic process for NSTI across seven tertiary centers, with the aim of determining the sensitivity, specificity, positive and negative predictive values, and accuracy of the method for diagnosing NSTI. This study represents an important step toward generating higher-quality evidence on the use of fluorescence imaging in patients with NSTI and its potential implementation in future clinical practice [24]. 

This systematic review’s main strength is that it is conducted in accordance with the PRISMA guidelines and the Cochrane methodology. Two researchers assessed the literature, performed study selection, and data extraction. The main limitation is the lack of high-quality studies identified, with only two case studies addressing the research question at hand. 

## Conclusions

This systematic review found a limited number of studies on ICG fluorescence-guided surgery in patients with NSTI, and the identified evidence was insufficient to draw firm conclusions. The two included case reports indicated the method's feasibility for NSTI, with a potentially better assessment of tissue perfusion than with direct human vision. ICG fluorescence imaging to identify necrosis has been examined in other clinical settings with promising results, though further studies are urgently needed to assess the role of ICG-guided surgery in patients with NSTI.
